# STRENGTHENING DEMENTIA CAREGIVING NETWORKS WITH TECHNOLOGIES: INTERSECTIONS OF POLICY, RESEARCH, AND PRACTICE

**DOI:** 10.1093/geroni/igz038.1692

**Published:** 2019-11-08

**Authors:** Nicole Ruggiano, Lauren R Bangerter, Melinda Gruber, Hannah Luetke-Stahlman, Kelly A O Malley, Jennifer L Wolff

**Affiliations:** 1 University of Alabama, Tuscaloosa, Alabama, United States; 2 Mayo Clinic, Rochester, Minnesota, United States; 3 Lakeland Health, Joseph, Michigan, United States; 4 Cerner Corporation, Kansas City, Missouri, United States; 5 New England GRECC, Boston VA Healthcare System, Boston, Massachusetts, United States; 6 Johns Hopkins University, Maryland, United States

## Abstract

Both family caregivers and providers report numerous challenges to providing care for of adults with Alzheimer’s disease and related dementias (AD/RD). Many of these challenges stem from ineffective communication and coordination within the health and support service networks, including: (1) communication challenges posed by AD/RD patients; (2) communication barriers between caregivers and providers; and (3) communication barriers among providers across institutions. Caregivers increasingly rely on technology to fill gaps in their knowledge, identify social support, and learn how to execute more complex care demands. While technology has been suggested as an effective strategy for improving the quality of AD/RD care and support caregivers, the role that public policy and research can and should play in advancing the goal of such tech-based solutions is still emerging. This is especially true in respect to how technology can strengthen care networks for patients and caregivers who are racial/ethnic minorities and/or living in rural communities. This presentation will identify the role that past legislation (e.g. the Affordable Care Act, HITECH, Medicaid) has played, as well as the role that recent and proposed legislation (e.g. RAISE Caregiver Act, CARE Act, 21st Century Cures Act, BOLD Infrastructure for Alzheimer’s Act, Older American’s Act reauthorization) should play in strengthening the health, support networks that caregivers interact with to provide ongoing care. This presentation will also summarize the current state of research on caregiver technologies and discusses how future research and policy initiatives can promote the translation of tech-based interventions into everyday care settings.

